# La schistosomose dans les zones de faible endémicité, une maladie trop négligée. Le cas de *Schistosoma monsoni* au Brésil

**DOI:** 10.48327/mtsi.v4i2.2024.469

**Published:** 2024-04-29

**Authors:** Ricardo PEREIRA IGREJA

**Affiliations:** Faculté de médecine, Université fédérale de Rio de Janeiro

**Keywords:** Schistosomose, *Schistosoma mansoni,* Endémicité, Maladie tropicale négligée, Diagnostic, Rio de Janeiro, Brésil, Amérique du Sud, Schistosomiasis, *Schistosoma mansoni,* Low endemicity, Diagnosis, Neglected tropical disease, Rio de Janeiro, Brazil, South America, Esquistossomose, *Schistosoma mansoni,* Baixa endemicidade, Diagnostico, Doença tropical negligenciada, Rio de Janeiro, Brasil, América do Sul

## Abstract

La schistosomose humaine est une maladie parasitaire causée par une infection par des trématode du genre *Schistosoma.* La maladie touche principalement les populations démunies. Environ 800 millions de personnes sont exposées au risque de contracter l’infection et il s’agit d’un problème de santé publique dans les régions tropicales et subtropicales d’Afrique, d’Asie, des Caraïbes et d’Amérique du Sud. Au Brésil, la schistosomose est largement répandue. On estime que 1,5 million de personnes en sont infectées et plus de 25 millions vivent dans des zones à haut risque de transmission. Dans l’État de Rio de Janeiro existent certaines zones présentant une faible endémicité ou des foyers isolés de *Schistosoma mansoni.* La plupart des individus infectés présentent des infections bénignes. Des études réalisées dans des zones de faible endémicité à Rio de Janeiro ont montré que si l’on analysait uniquement les enfants d’âge scolaire à l’aide du test de Kato-Katz, la majorité de la population infectée ne bénéficierait jamais de diagnostic d’infection à *S. mansoni.* Dans les situations de faible endémicité, avec de faibles intensités d’infection, avec une morbidité réduite dans la population et les tranches d’âge les plus touchées, la schistosomose nécessite une approche diagnostique plus sensible (par exemple, dépistage par PCR plutôt que test de Kato), sinon de nombreuses personnes infectées resteront indécelables par le système de santé.

## Abstract (en)

Human schistosomiasis is a parasitic disease caused by an infection with trematodes of the genus *Schistosoma.* The disease mainly affects impoverished populations. Around 800 million people are exposed to the infection, which is a public health problem in the tropical and subtropical regions of Africa, Asia, the Caribbean and South America. In Brazil, *Schistosoma mansoni* is the only species that causes schistosomiasis and the disease is widely distributed. Conventional diagnosis of the disease is carried out by detecting eggs using parasitological methods, such as the Kato-Katz test. Schistosomiasis has been reported in all regions of Brazil and is characterized as endemic in seven states in the Northeast Region and two states in the Southeast Region. In 2015, 78,7% of all cases reported in Brazil occurred in the Northeast Region. It is estimated that 1,5 million people is infected with this disease in Brazil and more than 25 millions live in areas with a high risk of transmission. Despite the reduction in mortality and morbidity, schistosomiasis was responsible for 8,756 deaths between 2000 and 2011 and 2,517 deaths between 2015 and 2019 in Brazil and it remains an important public health problem. In the state of Rio de Janeiro, some areas have low endemicity or isolated foci of *Schistosoma mansoni* and the majority of infected individuals have mild infections. The last survey of the disease in the state of Rio de Janeiro was carried out between 2010 and 2015 in students aged 7 to 17.

Schistosomiasis was reported in 10 of the 21 municipalities studied. Of the 5,111 school children screened for *S. mansoni* infection, 46 (1,65%) were tested positive. Studies carried out in areas of low endemicity in Rio de Janeiro showed that among the 205 patients infected by *S. mansoni* in Sumidouro, around 84% were aged 14 or over and all, except one individual, had the intestinal form (91,2%) or hepato-intestinal (8,3%) of schistosomiasis. Another study carried out in Sumidouro showed that with tests based on patent *Schistosoma* egg infection determined by the Kato-Katz test, active infections were diagnosed in eight (8/108) individuals. The intensity of infection expressed by parasite loads ranged from 6 to 72 eggs per gram of feces/individual. The results showed DNA amplification in 32 of the 100 individuals tested by real-time PCR. All individuals with patent ovo infection showed positive DNA amplification. These studies showed that if we only analyzed school-age children using the Kato-Katz test, the majority of the infected population would never be diagnosed with *S. mansoni* infection. In situations of low endemicity, with low intensities of infection, with low severity in the population and in the most affected age groups, schistosomiasis requires a more sensitive diagnostic approach (e.g. screening by PCR rather than Kato test), otherwise many infected individuals will remain invisible to the healthcare system.

## Abstract (pt)

A esquistossomose humana é uma doença parasitária causada por uma infecçâo por vermes sanguíneos do gènero *Schistosoma.* A doença afeta principalmente populaçoes empobrecidas. Cerca de 800 milhoes de pessoas estâo expostas à infecçâo, sendo um problema de saúde pública nas regioes tropicais e subtropicais de África, Ásia, Caribe e América do Sul. No Brasil, o *Schistosoma mansoni* é a única espécie causadora da esquistossomose e a doença é amplamente distribuida. O diagnóstico convencional da doença é realizado pela detecçâo dos ovos através de métodos parasitológicos, como o teste de Kato-Katz. A esquistossomose foi notificada em todas as regioes do Brasil, e é caracterizada como endèmica em sete estados da Regiâo Nordeste e dois estados da Regiâo Sudeste. Em 2015, 78,7% de todos os casos notificados no Brasil ocorreram na Regiâo Nordeste. Estima-se que 1,5 milhâo de pessoas estejam infectadas com esta doença no Brasil e mais de 25 milhoes vivam em áreas com alto risco de transmissâo. Apesar da reduçâo da mortalidade e morbidade, a esquistossomose foi relatada em 8.756 mortes entre 2000 e 2011 e em 2.517 mortes entre 2015 e 2019 no Brasil e continua sendo um importante problema de saúde pública. No Estado do Rio de Janeiro, algumas áreas apresentam baixa endemicidade ou focos isolados de *Schistosoma mansoni* e a maioria dos individuos infectados apresenta infecçoes leves. O último levantamento da doença no Estado do Rio de Janeiro foi realizado entre 2010 e 2015 em estudantes de 7 a 17 anos. A esquistossomose foi relatada em 10 dos 21 municipios estudados. Das 5.111 crianças escolares triadas para infecçâo por *S. mansoni,* 46 (1,65%) testaram positivo. Estudos realizados em áreas de baixa endemicidade no Rio de Janeiro mostraram que dentre os 205 pacientes infectados por *S. mansoni* em Sumidouro, cerca de 84% tinham 14 anos ou mais e todos, exceto um individuo, tinham a forma intestinal (91,2%) ou hepato-intestinal (8,3%) da esquistossomose. Outro estudo realizado em Sumidouro, mostrou que testes baseados em infecçâo patente de ovo de *Schistosoma* determinada pelo teste de Kato-Katz, infecçoes ativas foram diagnosticadas em oito (8/108) individuos. A intensidade de infecçâo expressa pelas cargas parasitárias variou de 6 a 72 ovos por grama de fezes/individuo. Os resultados mostraram amplificaçâo do DNA em 32 dos 100 individuos testados por PCR em tempo real. Todos os indivíduos com infecçâo ovo-patente apresentaram amplificaçâo de DNA positiva. Tais estudos mostraram que se analisarmos apenas crianças em idade escolar pelo teste de Kato-Katz, a maioria da populaçâo infectada nunca seria diagnosticada com infecçâo pelo *S. mansoni.* Em situaçoes de baixa endemicidade, com baixas intensidades de infecçâo, com baixa gravidade na populaçâo e nas faixas etárias mais afetadas, a esquistossomose requer uma abordagem diagnóstica mais sensivel (por exemplo, triagem por PCR em vez do teste de Kato), caso contràrio, muitos individuos infectados permanecerâo invisiveis para o sistema de saúde.

## Introduction

La schistosomose humaine est une maladie parasitaire chronique et aigüe causée par une infection par des trématodes du genre *Schistosoma,* dont *S. haematobium, S. mansoni, S. japonicum, S. mekongi, S. intercalatum, S. guineensis.* Il s’agit d’une maladie tropicale négligée (MTN), un groupe de diverses maladies et affections qui touchent principalement les populations à faible revenu dans le monde entier [12] Cette maladie est un problème de santé publique dans les zones tropicales et subtropicales d’Afrique, d’Asie, des Caraïbes et d’Amérique du Sud. Environ 800 millions de personnes sont exposées au risque de contracter l’infection. On estime que 200 millions de personnes sont infectées par l’une des espèces de schistosomes responsables de la maladie [12, 13]. Dans le but d’éliminer la schistosomose en tant que problème de santé publique, l’OMS avait initialement fixé un objectif de couverture de 75 % du traitement des enfants d’âge scolaire [4]. Les enfants sont particulirement vulnérables et leur responsabilité dans la transmission est très élevée en raison de certains de leurs comportements. Dans les zones de faible endémicité, avec des patients à faible charge parasitaire, comme dans l’État de Rio de Janeiro, les méthodes traditionnelles de lutte contre la schistosomose, c’est-à-dire le traitement des enfants d’âge scolaire diagnostiqués par des méthodes parasitologiques, peuvent être insuffisantes pour un diagnostic réel de la situation, ce qui rend la maladie encore plus négligée [3].

## La schistosomose au Brésil

Au Brésil, *Schistosoma mansoni* est la seule espèce responsable de la schistosomose et la maladie est largement répandue. Le diagnostic conventionnel de l’infection à schistosomose intestinale repose sur la détection des œufs par des méthodes parasitologiques telles que le test de Kato-Katz. L’infection a été signalée dans toutes les régions et est considérée comme endémique dans sept États de la région du Nord-Est et dans deux États de la région du Sud-Est. En 2015, 78,7 % de tous les cas signalés au Brésil proviennent de la région du Nord-Est [14]. Bien que près de 10 à 12 millions de personnes au Brésil aient été infectées par *S. mansoni* dans les années 1970, les programmes de contrôle ont réussi à réduire la mortalité, la morbidité et la transmission associées au Brésil. Cependant, au tournant du siècle, il y avait encore environ 2,5 à 6,3 millions d’individus infectés au Brésil [7]. La dernière enquête sur la maladie, menée dans le pays de 2010 à 2015 auprès d’élèves de 7 à 17 ans, a montré une réduction significative des cas positifs de la maladie. On estime que 1,5 million de personnes sont infectées par cette maladie au Brésil et que plus de 25 millions vivent dans des zones à haut risque de transmission [9]. Malgré la réduction de la mortalité et de la morbidité, la schistosomose a été à l’origine de 8 756 décès entre 2000 et 2011 et de 2 517 décès entre 2015 et 2019 au Brésil où elle reste un problème de santé publique important [10].

## La schistosomose dans l'Etat de Rio de Janeiro

Dans l’État de Rio de Janeiro, certaines zones présentent une faible endémicité (prévalence de la schistosomose chez les enfants d’âge scolaire inférieure à 10 % selon les paramètres parasitologiques) où existent des foyers isolés de *S. mansoni* dans lesquels la plupart des sujets infectés présentent des infections bénignes, excrétant moins de 100 œufs/g de selles. Lors d’une enquête réalisée dans l’État de Rio de Janeiro de 2010 à 2015 auprès d’élèves de 7 à 17 ans, la schistosomose a été signalée dans 10 des 21 municipalités étudiées. Sur les 5 111 écoliers dépistés pour l’infection à *S. mansoni,* 46 (1,65 %) ont été testés positifs [9]. Sumidouro, un comté de l’État brésilien de Rio de Janeiro, est entouré par des collines et possède des cascades et des rapides constituant ses principales collections hydriques. Il existe également quelques vestiges de la forêt tropicale brésilienne au sommet des montagnes et dans de petites parties le long des rives des cours d’eau. Plusieurs études sur les parasitoses intestinales ont été réalisées dans les localités rurales de la commune de Sumidouro, zone d’endémie de la schistosomose à *S. mansoni,* entre 1960 et 1980. Ces travaux ont montré de fortes prévalences de parasitoses intestinales dans les populations locales, probablement causées par les conditions sanitaires locales extrêmement mauvaises [1]. En ce qui concerne la schistosomose, son diagnostic à Sumidouro est désormais rendu difficile par les faibles charges de *S. mansoni,* ce qui favorise la sous-estimation du nombre d’individus infectés. Par ailleurs, la situation épidémiologique de la région est rendue plus complexe par la présence du rongeur sauvage *Nectomys squamipes,* hôte naturel de *S. mansoni,* qui participe au cycle local de transmission [6]. Parmi les 205 patients infectés par *S. mansoni* à Sumidouro, environ 84 % étaient âgés de 14 ans ou plus et tous, sauf un, avaient une forme intestinale (91,2 %) ou hépato-intestinale (8,3 %) de schistosomose [8].

On peut s’attendre à une telle configuration en cas d’infections bénignes, bien que des formes cliniques graves de schistosomose puissent aussi être présentes même dans les zones de faible endémie. Concernant le sexe, 69,8 % étaient des hommes et 30,2 % des femmes. Bien que l’exposition professionnelle à la schistosomose concerne tout le monde, les hommes sont plus exposés car ils se chargent seuls du nettoyage des collections hydriques. La sensibilité du test Kato-Katz diminue considérablement lorsque les charges parasitaires sont faibles, ce qui sous-estime les infections légères caractérisées par un faible nombre d’œufs. L’absence d’excrétion des œufs interfère directement avec la sensibilité de l’examen microscopique des échantillons de selles, compromettant ainsi la détection d’infections de faible intensité dans les zones de faible endémie. En revanche, les tests de détection d’ADN diagnostiquent même les individus avec un faible nombre d’œufs d’helminthes excrétés dans les fèces. En outre, les tests ADN jouent un rôle potentiel en tant que marqueurs des réponses au traitement. Dans une autre étude réalisée à Sumidouro, des tests de Kato-Katz ont révélé des infections actives chez 8 individus sur 108 prélevés. L’intensité de l’infection exprimée par les charges parasitaires variait de 6 à 72 œufs/g de fèces. Les résultats ont montré une amplification de l’ADN chez 32 individus sur 100 testés par PCR en temps réel [3]. En se fondant sur la méthodologie traditionnelle, analysant uniquement les enfants d’âge scolaire à l’aide du test de Kato-Katz, la majorité de cette population ne connaîtrait jamais de diagnostic d’infection à *S. mansoni*.

D’après l’OMS, il existe de bonnes preuves pour soutenir la poursuite de l’utilisation des outils conventionnels tels que le Kato-Katz et le Point-of-Care-CCA rapid test (POCCCA) pour détecter *S. mansoni* au cours de la surveillance épidémiologique dans les zones de prévalence modérée ou élevée, malgré la faible sensibilité pour les infections de faible intensité [11]. Cependant, des travaux supplémentaires sont nécessaires pour caractériser la sensibilité et la spécificité des outils de diagnostic immunologique et moléculaire de la schistosomose humaine. Toujours d’après l’OMS, de nouveaux outils de diagnostic sont nécessaires de toute urgence pour le diagnostic des schistosomes [12].

Dans une autre étude réalisée au Brésil, dans l’État de Minas Gerais, les tests parasitologiques et le test POC-CCA ont facilement détecté les individus présentant des infections lourdes ou modérées. En revanche, tous les tests ont montré des sensibilités réduites lorsqu’il fallait détecter des individus présentant une charge parasitaire faible (99-12 œufs/g de fèces) ou très faible (moins de 12 œufs/g de fèces). Surtout dans le cas d’une charge parasitaire très faible, la technique Kato-Katz, au mieux, n’a détecté que 40 *%* des individus infectés. Dans le cas des individus à très faible charge parasitaire, les méthodes POC-CCA et Helmintex ont montré de meilleures performances avec des sensibilités de plus de 50 et 84 % respectivement. Cependant, dans sa forme actuelle, Helmintex n’est pas applicable à grande échelle en raison de la taille de l’échantillon requise et des processus de tamisage et de sédimentation qui prennent beaucoup de temps. Ce test pourrait cependant constituer une référence pour l’évaluation de nouveaux outils de diagnostic développés pour le terrain. En recherchant des méthodes de diagnostic facilement applicables avec une sensibilité améliorée pour les études épidémiologiques, il a été constaté que la combinaison de la méthode Kato-Katz avec le POC-CCA produisait de meilleurs résultats lorsque le nombre de lames et d’échantillons fécaux était augmenté [11].

En revanche, Ferrer *et al.* ont montré que l’ELISA basé sur l’antigène soluble de l’œuf (ELISA-SEA) est la meilleure méthode pour le diagnostic des infections actuelles et anciennes et que la PCR sur les fèces est la méthode la plus efficace pour détecter une transmission récente [5]. L’utilisation de différents tests complémentaires a également permis un meilleur diagnostic de l’infection à *S. mansoni,* révélant une prévalence relativement élevée (33,8 %) de la schistosomiose dans une communauté de faible transmission au Venezuela. La technique Mini-FLOTAC représente une technique fiable pour détecter *S. mansoni* zoonotique et d’autres parasites dans les réservoirs de rongeurs [2].

Une combinaison de méthodes doit être mise en œuvre puisque les programmes de lutte contre la schistosomose dans différentes régions du monde évoluent, en passant du contrôle de la morbidité vers le contrôle de la transmission et l’élimination [11].

## Le contrôle de la schistosomose

La schistosomose est incluse comme priorité dans les agendas mondiaux tels que les objectifs de développement durable (ODD) et la feuille de route pour le contrôle des MTN de l’Organisation mondiale de la santé (OMS). Actuellement, la lutte contre la schistosomose dans les régions endémiques repose sur l’administration en campagne à large échelle régulière de médicaments et la surveillance des infections chez les enfants d’âge scolaire. En 2020, l’OMS a publié une nouvelle feuille de route pour guider l’action contre les MTN au cours de la décennie 2021-2030. Cette feuille de route vise l’élimination de la schistosomose en tant que problème de santé publique et l’interruption de la transmission du schistosome chez les humains dans certains pays d’ici 2030. La stratégie de l’OMS pour contrôler et éliminer la schistosomose humaine comprend la chimiothérapie préventive des groupes à risque, l’accès à une source d’eau potable et à un système d’assainissement amélioré, l’éducation à l’hygiène, la gestion de l’environnement et la lutte contre les mollusques réservoirs [13]. La maladie touche principalement les populations démunies qui, soit ignorent le potentiel de transmission des sources d’eau qu’ils utilisent, soit ne peuvent éviter le contact avec de l’eau infestée en raison de leur profession (agriculture, pêche), de leurs comportements récréatifs (nager et jouer dans l’eau) ou du manque d’une source fiable d’eau potable.

## Conclusion

À tout âge, le contact avec des cercaires d’eau douce peut entraîner une infection à schistosomes. En effet, la schistosomose touche les populations pauvres du monde, qui dépendent généralement des plans d’eau locaux et risquent d’y être exposées, parfois quotidiennement [4]. Dans les situations de faible endémicité, avec de faibles charges parasitaires, ce qui d’une part, entraîne une perte d’efficacité des outils diagnostiques et d’autre part, augmente le désintérêt général pour cette maladie déjà négligée, la maladie nécessite une approche diagnostique différenciée par rapport aux zones d’endémicité moyenne et élevée. L’utilisation de techniques de laboratoire d’une plus grande sensibilité, combinée à un élargissement de la population étudiée, est essentielle pour un diagnostic réel de la situation dans les zones de faible endémicité.

## Approbation éthique

N’est pas applicable.

## Financement/Soutien

Aucun.

## Liens d'intérêts

L’auteur ne déclare aucun conflit d’intérêt
